# Aberrant functional connectivity in depression as an index of state and trait rumination

**DOI:** 10.1038/s41598-017-02277-z

**Published:** 2017-05-19

**Authors:** David Rosenbaum, Alina Haipt, Kristina Fuhr, Florian B. Haeussinger, Florian G. Metzger, Hans-Christoph Nuerk, Andreas J. Fallgatter, Anil Batra, Ann-Christine Ehlis

**Affiliations:** 1 Department of Psychiatry and Psychotherapy, University Hospital of Tuebingen, Tuebingen, Germany; 2 Center of Integrative Neuroscience (CIN), Cluster of Excellence, University of Tuebingen, Tuebingen, Germany; 3 Department of Psychology, University of Tuebingen, Tuebingen, Germany; 4 LEAD Graduate School and Research Network, University of Tuebingen, Tuebingen, Germany; 5 Geriatric Center, University Hospital Tuebingen, Tuebingen, Germany; 6 Leibniz-Institut für Wissensmedien, Tuebingen, Germany

## Abstract

Depression has been shown to be related to a variety of aberrant brain functions and structures. Particularly the investigation of alterations in functional connectivity (FC) in major depressive disorder (MDD) has been a promising endeavor, since a better understanding of pathological brain networks may foster our understanding of the disease. However, the underling mechanisms of aberrant FC in MDD are largely unclear. Using functional near-infrared spectroscopy (fNIRS) we investigated FC in the cortical parts of the default mode network (DMN) during resting-state in patients with current MDD. Additionally, we used qualitative and quantitative measures of psychological processes (e.g., state/trait rumination, mind-wandering) to investigate their contribution to differences in FC between depressed and non-depressed subjects. Our results indicate that 40% of the patients report spontaneous rumination during resting-state. Depressed subjects showed reduced FC in parts of the DMN compared to healthy controls. This finding was linked to the process of state/trait rumination. While rumination was negatively correlated with FC in the cortical parts of the DMN, mind-wandering showed positive associations.

## Introduction

In the last decade, the study of aberrant functional and structural connectivity in depression has become a promising endeavor for the understanding of maladaptive processes underlying its psychopathology. Functional connectivity (FC) is defined by the functional co-activation of spatially distributed brain regions^1^. The analysis of FC in resting-state and task conditions has revealed aberrant function in various brain networks in Major Depressive Disorder (MDD), both in early life as well as in late-life depression (LLD)^2–4^. However, until today the corresponding psychopathological processes that are associated with aberrant FC in MDD are unexplained. The present study aimed at clarifying the processes that are related to alterations in FC in MDD.

Higher FC in MDD and LLD in parts of the Cognitive Control Network (CCN) and the Default Mode Network (DMN) have often been interpreted as manifestations of depression-specific processes^5, 6^. Especially the DMN – which anatomically consists of the precuneus, adjacent posterior cingulate/retrospinal cortex, the inferior parietal lobe/AngG (angular gyrus) and the medial prefrontal cortex^7^ – has been proposed to play a role in depressive rumination, due to its importance for self-referential processes.

Although there is no unifying definition of depressive rumination^8^ it can roughly be defined as a repetitive, rather abstract style of thinking that is focused on the past or shortcomings of oneself. The interpretation of abnormal FC in MDD as a neural correlate of rumination is rather appealing, since rumination is associated with the severity of MDD in regards to duration, symptom severity, risk for suicide, risk for relapse and cognitive functioning^8–12^. However, the evidence that altered FC in MDD reflects depressive rumination is heterogeneous^13–16^. Also, studies vary in their FC measurement, including measurements of “spontaneous” and “induced” rumination.

Regarding induced rumination, there are some limitations that make it difficult to compare or generalize effects. First, the induction of rumination (e.g., via recall of autobiographical information) may induce artificial or confounding neural activation unrelated to rumination per se, but to other aspects of the induction process, e.g. increased cognitive load. Another limitation pertains to the assessment of rumination. Most studies use trait-questionnaires, that measure rumination as a habitual reaction to sad mood. Thus, rumination is measured as a trait-construct and is correlated to a (state-) resting state measurement of FC. This leaves the possibility that patients with high trait rumination actually are not ruminating during the resting state measurement. The reported correlation between rumination and FC could then be attributed to a trait construct of depression (e.g. neuroticism) rather than to the state process of rumination.

Therefore, the main goal of this study was to investigate state and trait contributions of rumination to altered FC measures in depressed patients and healthy controls using functional near-infrared spectroscopy (fNIRS). To explore the unconstrained flow of ruminative thought, we used a quasi-experimental approach that combined qualitative and quantitative measures. To assess trait- and state-aspects of rumination, we used the rumination response scale (RRS) and visual analogue scales (VAS) after the resting-state measurements respectively^17^. Additionally, subjects were asked to describe their inner experiences during the resting-state measurement in detail on a blank page – the self-report form. We hypothesized that depressed subjects would report more ruminative thinking and less mind-wandering during resting-state, and show a higher level of trait rumination than non-depressed subjects. Regarding FC measurements, we expected both state and trait rumination to be anti-correlated with FC in regions of the parietal cortex.

## Results

**Figure 1:**
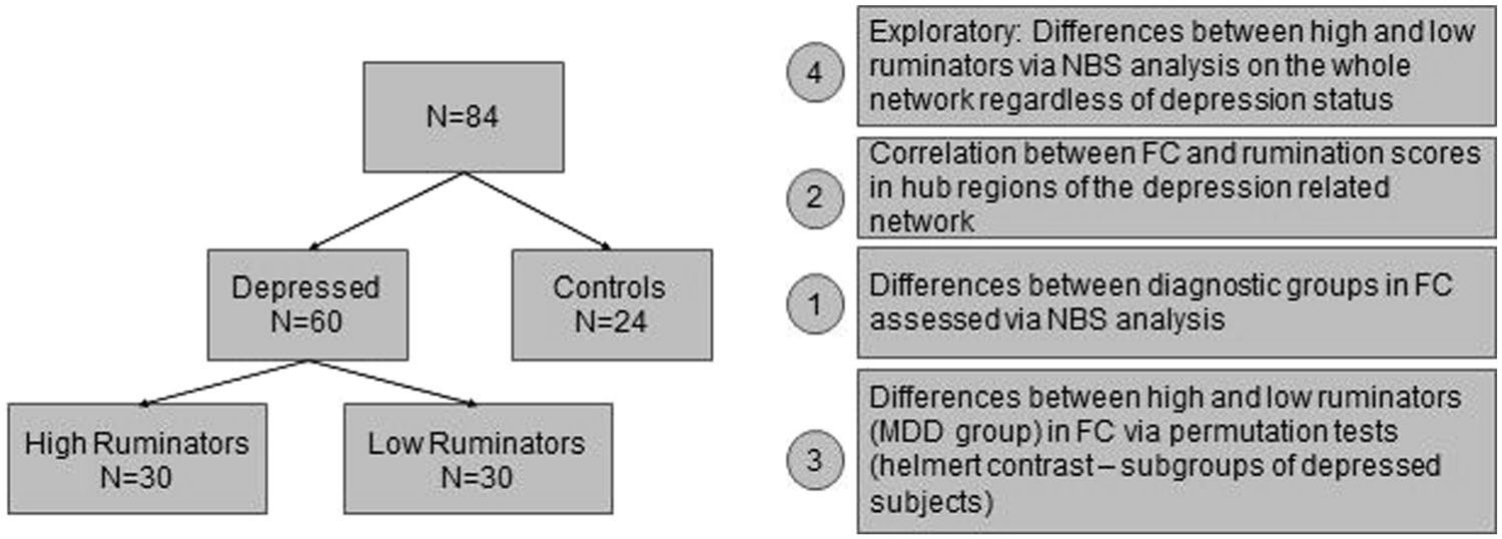
Analysis scheme: Analysis steps 1, 2 and 4 were performed on the whole sample. In the third analysis step, only the depressed subjects were investigated.

The following analysis was performed on the data: After the computation of FC measures, network-based statistics (NBS) were used to identify network-differences in FC between depressed and non-depressed subjects. Afterwards, the effects of state and trait rumination on these differences were assessed by using these variables as covariates in the NBS-model. For further illustration of the effects of rumination, hub nodes of the depression-related network were used as seed regions for further analysis: First, correlations between the FC to these hubs and the rumination scores were computed and plotted for the whole sample. Since depression status and rumination may be confounded and the correlation between rumination and FC in the whole sample might be spurious (because of between-group differences in both of these variables), we also performed a subgroup analysis by separating the depressed subjects into a high rumination and low rumination group as defined by median split of the rumination scales. Differences in FC in the hub nodes between these two sub-groups were assessed via permutation tests using maximal statistic^18, 19^. Finally, the main effects of state and trait rumination on FC were analyzed by deriving network differences via NBS for high and low ruminators for the whole sample. This analysis step was used for an exploratory investigation of the network organization of low and high ruminators to better understand the overlap between the effects of depressive status and rumination. Figure 1 shows an overview over the analytical steps.

Qualitative: 80 subjects (95%) listed at least one of the following categories in their self-report form: mind-wandering (59.5%), future things to do/making plans (40.5%), fighting against fatigue (38.1%), rumination (31%), thinking about the measurement itself and the instructions (20.2%), suppressing inner experiences (16.7%), thinking about the duration of the measurement (16.7%), doing active relaxation – e.g. mindful focus (15.5%), feeling body sensations (14.3%), hearing sounds, e.g. the NIRS machine (8.3%), feeling bored (4.8%). The healthy controls (HC) described significantly more focus on body sensations (29.2% of HC vs. 8.3% of the patients; χ²_(1)_ = 6.076, p < 0.05, OR = 0.221), more focus on external sounds (33.3% vs. 8.3%; χ²_(1)_ = 8.191, p < 0.01, OR = 0.182), more mind wandering (87.5% vs. 48.3%; χ²_(1)_ = 10.915, p < 0.001, OR = 0.134) and less rumination (8.3% vs. 40%; χ²_(1)_ = 8.044, p < 0.01, OR = 7.33).

On the resting-state scales, depressed subjects showed higher levels of state rumination (t_(82)_ = 3.64, p < 0.001, d = 0.83), lower levels of mind-wandering (t_(82)_ = 2.445, p < 0.05, d = 0.58) and lower levels of focus on sensations (t_(82)_ = 2.831, p < 0.01, d = 0.72). The groups also differed in their trait rumination (t_(82)_ = 8.406, p < 0.001, d = 2.0). Trait rumination was negatively correlated with mind-wandering (r_(82)_ = −0.42, p < 0.001) and positively correlated with state rumination (r_(82)_ = 0.32, p < 0.001). State rumination was negatively correlated with mind-wandering (r_(82)_ = −0.50, p < 0.001) and focus on sensations (r_(82)_ = −0.37, p < 0.001) (Table 1).

**Table 1:** Pearson correlations between the resting-state scales and trait rumination.

	RRS	Scale Rumination-state	Scale FAF	Scale Mind-Wandering	Scale Body
RRS	1				
Scale Rumination-state	0.32**	1			
Scale FAF	0.18	−0.10	1		
Scale Mind-Wandering	**−0.42** ^**^	−0.50**	−0.40**	1	
Scale Body	−0.02	−0.37**	−0.28*	−0.22*	1

N = 84, *p < 0.05, **p < 0.001.

### Differences between HC and patients

**Figure 2:**
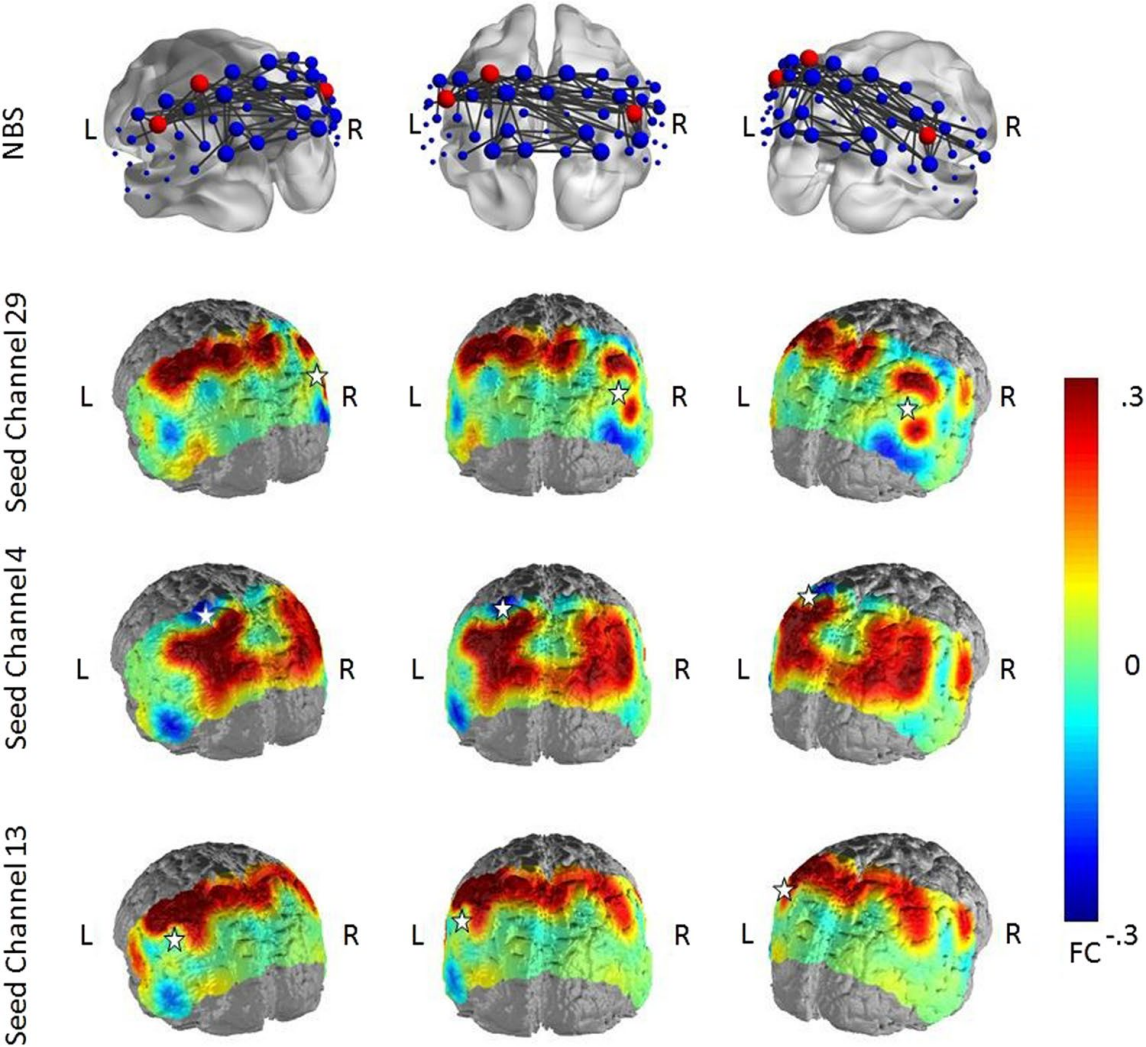
Differences between non-depressed and depressed subjects in FC in the NBS analysis at t = 2.7 and in selected seed regions (red nodes in the network maps). Warm colors indicate higher FC in the non-depressed subjects. Seed regions are marked by a white star.

The NBS analysis of differences in FC between depressed patients and HC revealed significant network disconnection in the depressed group at all statistical thresholds (Table 2). Depending on the statistical threshold (t_(82)_ = 2.7 to t_(82)_ = 3.4), the derived disconnected network consisted of 36 to 8 nodes with 72 to 8 edges (p = 0.003 ± 0.0015 to p = 0.016 ± 0.0035). The disconnected network was bilaterally organized within regions of the DMN and consisted mainly of interhemispheric FC differences. In the same way, hub nodes were consistently localized within cortical regions of the DMN: the middle somatosensory association cortex (SAC), left supramarginal gyrus (SupG) and right AnG (Fig. 2). Effect sizes in the three seed regions ranged between d = 0.90 to 0.47 in the left SupG, d = 0.81 to 0.39 in the middle SAC and d = 0.81 to 0.64 in the right AnG. Note that, when placing seeds, some regions with higher FC appeared for the depressed group, lying outside the cortical parts of the DMN and not being part of the NBS cluster solution.

**Table 2:** Degrees of the significant network differences between Depressed and Non-Depressed subjects at t_(82)_ = 2.7, t_(82)_ = 3.0 and t_(82)_ = 3.4.

Channel	Region	Depressed vs. Non-Depressed		
		t = 2.7	t = 3.0	t = 3.4
		Degree	Degree	Degree
2	SupG	4	2	1
3	SupG	6	**5**	**3**
**4**	**SAC**	**10**	**5**	**2**
5	SAC	6	**5**	—
6	SAC	7	4	—
7	SAC	3	2	—
8	SupG	3	2	—
10	SA	2	1	—
12	SupG	1	1	—
**13**	**SupG**	**9**	**6**	**2**
14	AngG	3	1	—
15	SAC	8	3	—
16	SAC	5	3	—
17	SAC	2	2	—
18	SupG	5	2	2
19	SupG	3	2	—
20	PSC	1	—	—
21	STG	3	1	—
23	SupG	1	—	—
24	AngG	1	1	—
25	SAC	1	—	—
26	SAC	1	—	—
28	SAC	2	2	—
**29**	**AngG**	**9**	**5**	**3**
30	SupG	1	1	—
35	SAC	2	1	1
36	SAC	5	4	—
38	V3	7	5	—
39	AngG	1	—	—
40	AngG	7	**7**	**2**
45	AngG	1	—	—
46	V3	7	3	—
47	V3	6	**5**	—
48	V3	2	1	—
49	V3	8	4	—
50	AngG	1	—	—
nodes		36	29	8
edges		72	43	8
p-value		0.003 ± 0.0015	0.003 ± 0.0015	0.016 ± 0.0035

Only channels of the significant network are presented. SAC = somatosensory association cortex, SupG = supramarginal gyrus, AngG = angular gyrus, STG = superior temporal gyrus, FusG = fusiform gyrus, MTG = middle temporal gyrus, PSC = primary somatosensory cortex, SC = subcentral area. Bold numbers are hub nodes.

### Differences between HC and patients when controlled for rumination

When controlling for state rumination, the significant network differences between depressed and non-depressed subjects were reduced at all statistical thresholds (t_(81)_ = 2.7, p = 0.010, nodes = 29, edges = 50; reduced by 7 nodes and 43 edges; t_(81)_ = 3.0, p = 0.034, nodes = 11, edges = 12; reduced by 18 nodes and 31 edges; t_(81)_ = 3.4, p = 0.041, nodes = 7, edges = 6; reduced by 1 node and 2 edges). Over all three thresholds, FC was reduced due to the covariate mostly in the middle SAC (Channel 4,5,6,16) and in V3 (Channel 38,46,49).

At all statistical thresholds, the network differences between depressed and non-depressed subjects did not reach significance when controlled for trait rumination. Remarkably, this means that no significant variance in FC could be explained by depression status when controlled for trait rumination.

### Correlations of rumination and FC in the depression-related network

**Figure 3:**
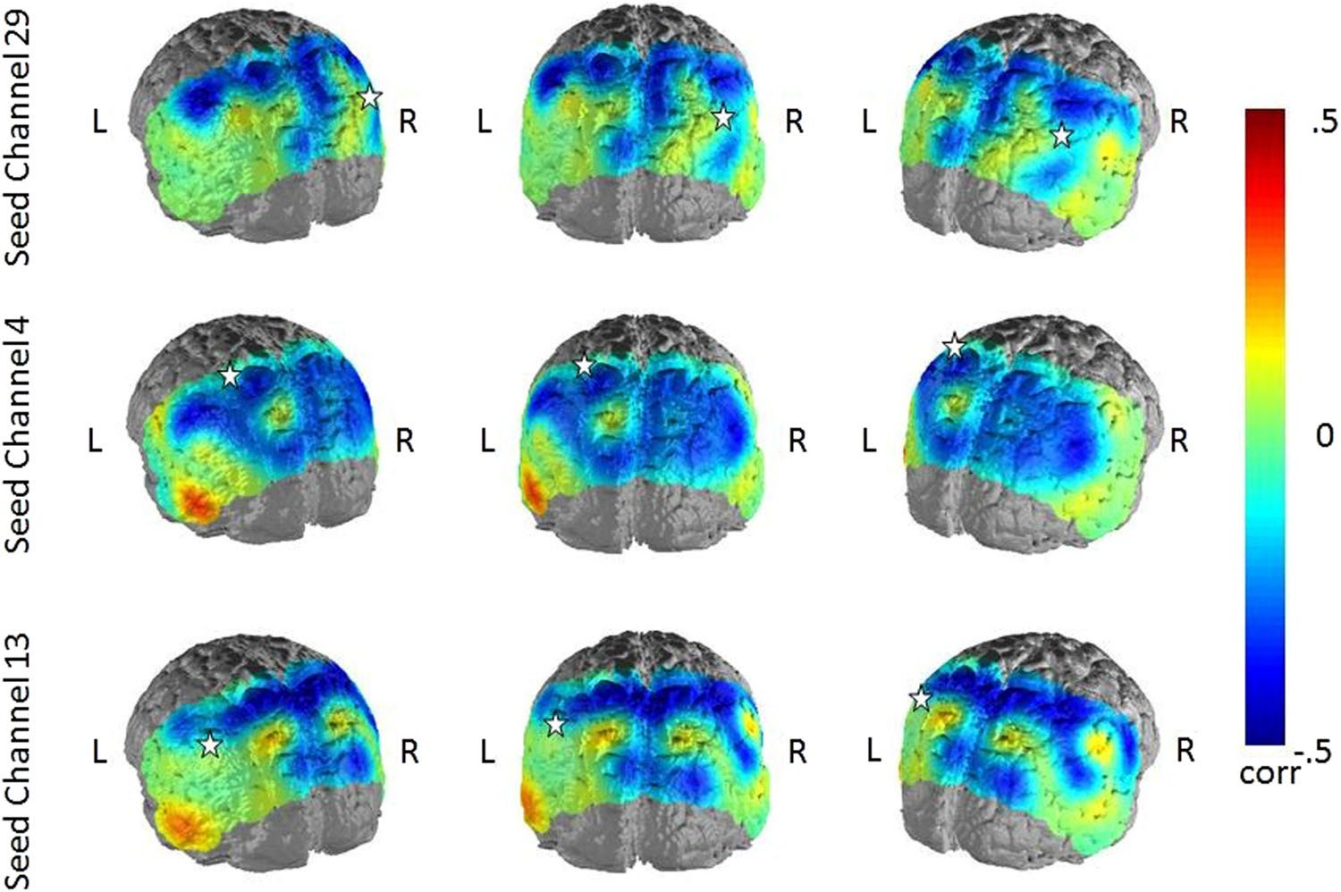
Correlations between trait rumination and FC in the three seed regions of the depression-related network. Seed regions are marked by a white star.

**Figure 4:**
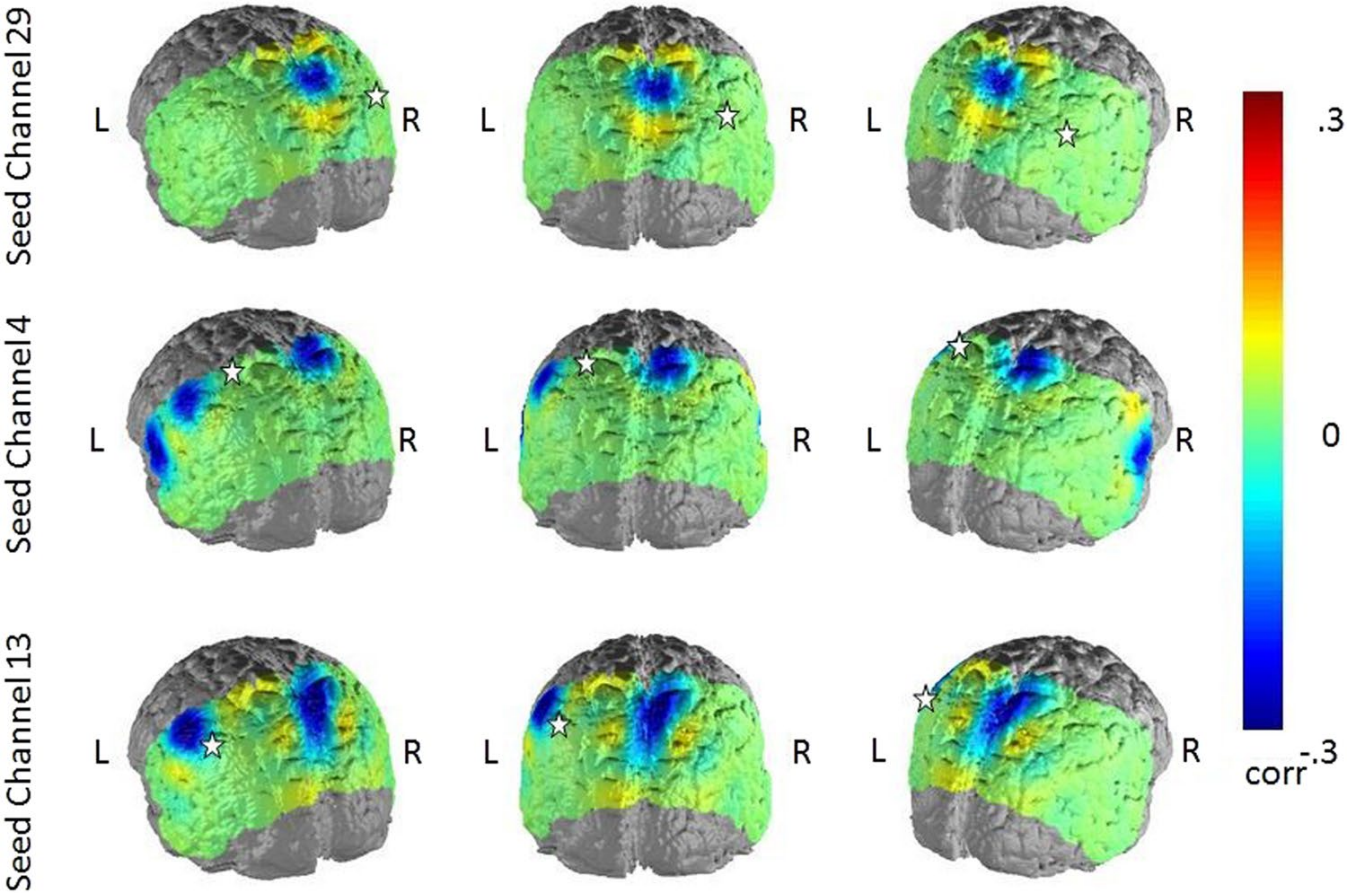
Correlations between state rumination and FC in the three seed regions of the depression-related network. Seed regions are marked by a white star.

When correlating the scores of trait and state rumination with the FC-scores to the defined seed regions of the depression-related network, we observed for both variables a negative relationship with FC (Figs 3 and 4). The association between trait rumination and FC was higher and more wide-spread over the whole posterior probeset in all three hub nodes, ranging from −0.36 to −0.22 (p < 0.001 to p < 0.05) for the seed region in the right AnG, from −0.36 to −0.21 (p < 0.001 to p < 0.05) in the SAC and from −0.42 to −0.23 (p < 0.001 to p < 0.05) in the left SupG. From these, only correlations with a size > 0.31 survived correction for multiple comparisons. The correlations between state rumination and FC were also negative but weaker and more focused in their distribution ranging between −0.29 to −0.22 (p < 0.01 to p < 0.05) for the seed region in the left SupG and between −0.28 and −0.25 in the middle SAC (p < 0.01 to p < 0.05). However, none of the correlations remained significant after controlling for multiple comparisons. For the right AnG, only the FC to the middle SAC showed a negative relationship to state rumination (rho = 0.−26, p < 0.01). For the two remaining seed regions, associations between state rumination and FC were mainly restricted to this area and the left SupG and AnG. As for the FC differences between depressed and non-depressed subjects, spurious positive correlations between trait rumination and FC from the seed regions to regions outside the DMN were observed.

### Depressed Ruminators vs. Depressed Non-Ruminators

**Figure 5:**
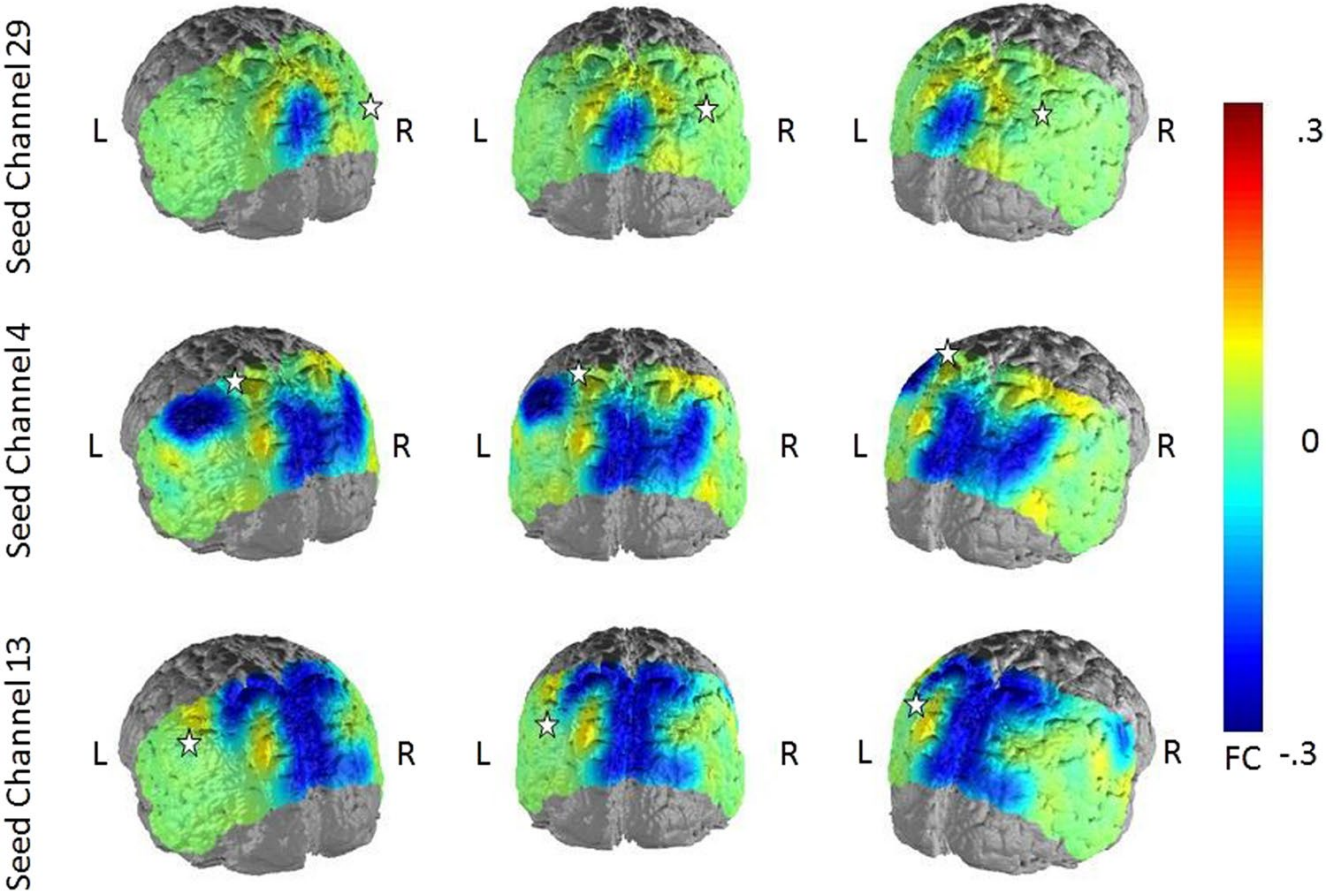
Differences between “depressed low trait-ruminators” and “depressed high trait-ruminators”. Cold colors indicate lower FC in high-ruminators compared to low-ruminators.

To investigate whether the results in the previous section were only due to differences between diagnostic groups on both FC and rumination variables, we performed a subgroup analysis for “depressed high ruminators” and “depressed low ruminators”. Following a median split for state and trait rumination in the depressed sample, we compared the FC in the depression-related network to the three seed regions for the subgroups by performing permutation tests. Like in the correlation analysis of the whole sample, again trait rumination showed a stronger association with FC than state rumination.”Depressed high trait-ruminators” showed reduced FC compared to the “depressed low trait-ruminators” comparing all three seed regions (Fig. 5). Effect sizes ranged between d = −0.39 to −0.66 for the seed region in the SAC, d = −0.40 to −0.90 in the left SupG and was d = −0.60 in the seed region of the AngG regarding the FC to the middle SAC and V3. In contrast to the correlation analysis, significant differences (p < 0.05) in FC between these rumination groups were focused to regions in the middle SAC and left SupG.

**Figure 6:**
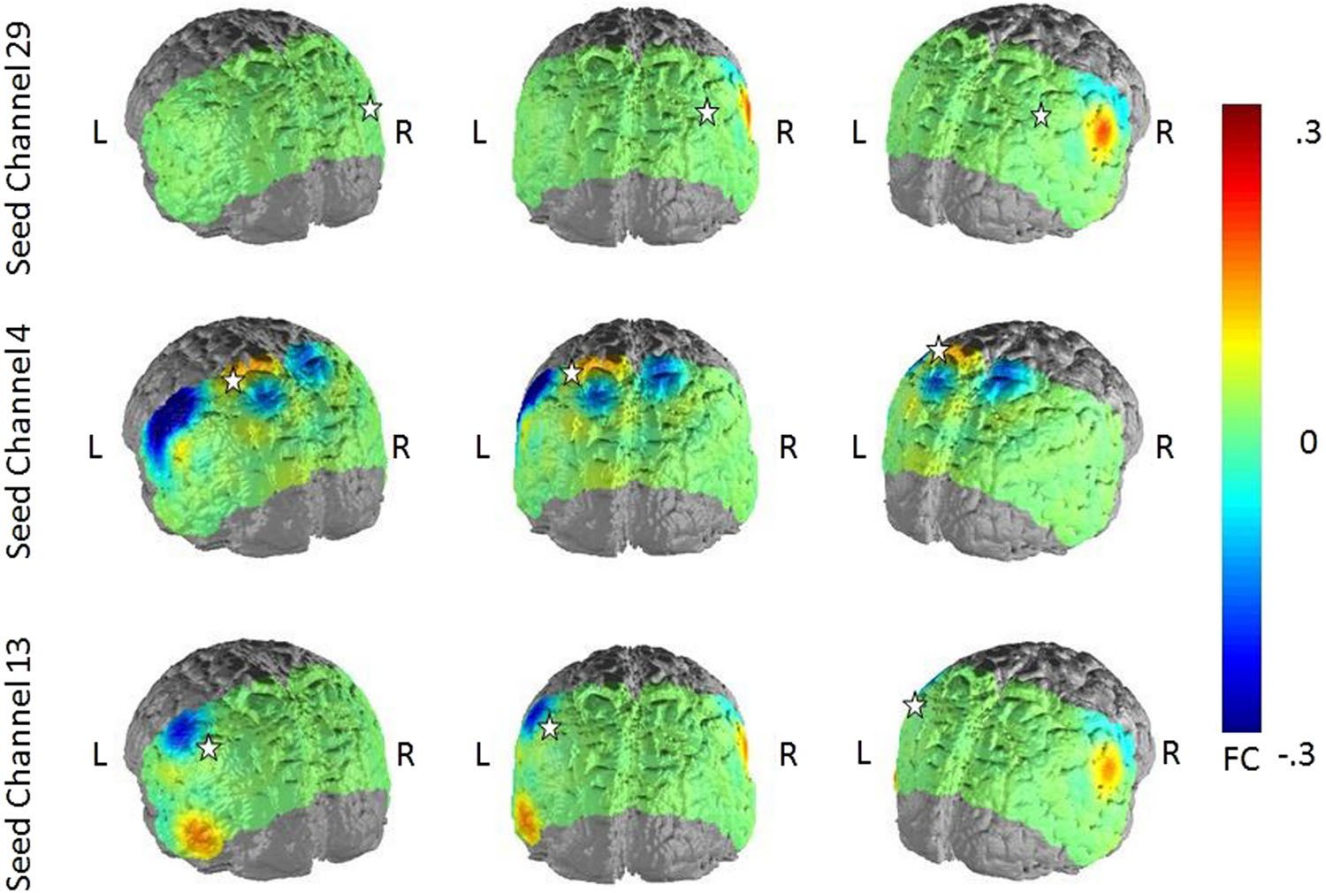
Differences between “depressed low state-ruminators” and “depressed high state-ruminators”. Cold colors indicate lower FC in high-ruminators compared to low-ruminators.

Differences between “depressed high state-ruminators” and “depressed low state-ruminators” were only significant (p < 0.05) in the seed regions of the left SupG and middle SAC. Significant differences in FC were also located in the middle SAC and left SupG (Fig. 6). Effect sizes for the seed region of the middle SAC ranged between d = −0.34 and −0.68 and were d = −0.40 for the seed region in the left SupG. In the latter seed regions, higher FC was also observed in the left middle temporal gyrus (d = 0.41) and right primary somatosensory cortex (d = 0.46) for the “depressed high-state ruminators”, which was consistent with the correlation analysis of trait rumination and the NBS analysis of depressed and non-depressed subjects.

### Main effects of rumination

For a better interpretation of the results reported above, we also ran an exploratory analysis via NBS for the main effects of state and trait rumination regardless of the depression status to reveal differences in FC outside the depression-related network. Both, state and trait rumination revealed a significantly disconnected network for “high ruminators”. The disconnected network for trait rumination consisted of 37 nodes and 87 edges (p = 0.002 ± 0.0013) with hub nodes in the middle SAC and V3. The network showed a bilateral organization with dense disconnections in the regions of the DMN – namely the middle SAC and the left and right SupG and AngG (Figure S4). Effect sizes for the seed region in the middle SAC (Channel 16) ranged between d = −0.38 to d = −0.79.

The state rumination related disconnected network comprised 21 nodes and 29 edges (p = 0.022 ± 0.0041) with hub nodes in the middle SAC and the left SupG (Figure S5). The network showed a left hemispheric focus with dense disconnections between the middle SAC and the left SupG and left AngG. Effect sizes for the seed region in the middle SAC ranged between d = −0.33 to d = −0.81.

## Discussion

The aim of this study was to investigate the impact of state and trait rumination on differences in FC between depressed and non-depressed subjects. Our qualitative measurements revealed that depressed subjects ruminated more than non-depressed subjects. However, only 40% of the depressive sample reported ruminative content, and state and trait rumination were only moderately correlated, suggesting independent constructs. Both state and trait rumination showed strong anti-correlations with the process of mind-wandering – one of the hypothesized core processes behind the DMN.

As expected from our previous findings^6^ and the observed anti-correlation between CCN and DMN^20^, we found reduced FC within regions of the DMN in the depressed sample compared to the non-depressed sample. These findings are in line with other studies that found disrupted FC in MDD between posterior and temporal areas^21^, posterior cortex and bilateral caudate^22^, in inter-hemispheric FC^23^, in the salience network^24^ and between functional connectivity networks^25^. In our study, FC to seed regions in the depression-related network were anti-correlated to state and trait rumination. These effects stayed stable when running a subgroup analysis of “high state-/trait-ruminators” vs. “low state-/trait-ruminators” within the depressed sample only. The effects of trait rumination on FC in the seed regions were stronger and more widespread than the effects of state rumination. A possible explanation for this variation in the strength and (spatial) extent of effects might lie in the constructs themselves: while state rumination is a rather narrow process and construct, trait rumination is a much more broadly defined concept that might be linked to other constructs such as neuroticism or distractibility which in turn might influence FC^8^. However, both state and trait rumination showed associations to FC differences in the depression-related network and may therefore explain differences in FC between depressed and non-depressed subjects.

When examining the main effects of state rumination on FC in the whole probeset (and not only in the depression-related network), it became clear that the disconnected network for the “high state-ruminators“ had a left-hemispheric focus with hub nodes in the left SupG und middle SAC. Interestingly, the left hemispheric focus of the effects of state rumination on FC is consistent with our previous findings^6^. This effect might be due to specialization of the hemispheres^26^. In contrast, the effects of trait rumination showed a much broader distribution over the cortical DMN as indicated by a bilaterally organized network with dense connections between the DMN nodes. However, both state and trait rumination showed effects similar in size and consistently in the middle SAC and left SupG and AnG.

As another implication, our results also indicate an anti-correlation between rumination and the process of mind-wandering. At this point, the question arises if the association between state rumination and FC is solely explained by this anti-correlation between state rumination and mind-wandering. From our point of view, the processes of mind-wandering and rumination are two sides of the same medal: Mind-wandering – as measured by our resting-state questionnaire – is defined as being in a relaxed state, in which a person’s thoughts flow in an unconstrained way without any focus on a particular subject. State rumination on the other hand is defined as a repetitive stressing style of thinking about unfinished concerns that leads to the urge of suppressing the inner experience. From this point, it becomes clear that a person cannot be in the process of mind-wandering and the process of rumination at the same time. This antagonistic relationship is reflected by the anti-correlation of the processes and the FC differences between the (high mind-wandering) non-depressed and the (high ruminating) depressed subjects. It would be an interesting attempt for future research to categorize and disentangle these different “styles of thinking”.

Regarding previous findings on FC in depression and rumination, our results are in line with studies reporting a negative association between FC in parietal parts of the DMN and rumination and disrupted network organization in MDD^13, 15, 16, 23, 25, 27, 28^. For example, Jacobs *et al*.^16^ found a negative association between a factor analysis derived factor in the PCC and trait rumination. In line with this, Berman *et al*.^15^ reported reduced global FC for depressed subjects, compared to healthy controls. However, in the same study elevated levels of FC were reported during induced rumination in MDD patients. Other studies also show a positive association between FC in the DMN and depression and rumination^14, 29–32^. For example, Cooney *et al*.^29^ found that rumination is associated with enhanced activity in OFC, DLPFC, rostral anterior cingulate, posterior cingulate and parahippocampus. Also, increased FC in the DMN is found during stages of induced rumination^33^. Since positive associations between FC and rumination in the DMN are also found during phases of spontaneous rumination, these effects cannot be fully attributed to artificially induced activation by induction tasks.

Here, our results seem to be in conflict with previous research. Interestingly, most studies that reported higher FC in depressed subjects found higher FC between sgACC and the PCC. Similarly, in our previous own work we identified enhanced FC between anterior and posterior regions of the CCN^6^. In their review of the fMRI literature regarding rumination and FC, Hamilton and colleagues (2015) argue that the often found positive correlation between sgPFC and the DMN reflects “a functional integration of properties of the sgPFC and DMN”. These functions include “imbuing of internal stimuli with valence” (DMN) and “affectively laden behavioral withdrawal” supported by the sgPFC^34^. Since rumination and its immanent withdrawal aspect are rather attention demanding processes, one might suggest that they are associated with enhanced FC between areas in the fronto-parietal networks supporting higher cognitive processes. Our results of reduced FC in MDD in the parietal cortex – including cortical parts of the DMN – might be just in line with this hypothesis and data. The parietal cortex plays a central role in the integration of sensory information. In the same way, the DMN is thought to play a central role in the integration of egocentric information. If a subject is in a mental state that uses such functions – such as mind-wandering – the parietal cortex and the cortical parts of the DMN show higher functional integration. However, if attention demanding states are present – such as during rumination – this functional integration of the parietal cortex should be interrupted. Instead, these cortex areas might then be demanded in other processes and show a high functional integration with anterior regions (like the DLPFC, sgPFC, ACC). The latter assumption is supported by a recent meta-analysis, showing hyper-connectivity between the fronto-parietal CCN and the DMN during resting-state^35^.

A second aspect concerns the bilateral organization of the derived network differences between depressed and non-depressed subjects and low and high trait ruminators. Most of the network differences in our study between these groups comprised inter-hemispheric differences. So far, there are several studies that show decreased inter-hemispheric FC in MDD^36–40^. However, the biological background of inter-hemispheric FC abnormalities is not fully understood, although studies from split brain patients suggest that a disruption of inter-hemispheric FC affects the information processing and functioning of the brain^41, 42^. In light of this work, one might argue that most of the cortical DMN differences in FC we found could be due to the reduced inter-hemispheric FC found in the MDD population. However, this interpretation does not account for the medial temporal disconnections and the left hemispheric focus of the state rumination network.

Aside from the promising and mostly conclusive findings reported above, some limitations have to be considered: Although fNIRS is a well-suited method to obtain neurophysiological data of hemodynamic changes in the cortex, its depth resolution is restricted to cortical structures and the covered area is restricted to the size of the used probeset. Therefore, with this method it is not possible to cover the DMN completely. However, we as others showed that fNIRS is suited to measure the cortical structures of the DMN. Moreover, Sasai *et al*.^43^ showed in a combined fNIRS/fMRI study that cortically measured fNIRS signals correlated not only with cortical fMRI signals, but also with subcortical parts of the brain networks^43^. However, as long as there is no co-registered fMRI measure, such subcortical projections can only be hypothesized from the imputation of fNIRS results. Although fMRI remains the gold standard in tracking hemodynamic changes in the brain, fNIRS may be the advantageous method in some cases due to its high time resolution, easy assessment in natural environments, relative robustness against movement artifacts and low operating costs.

Another limitation concerns the difference in age between the groups. The depressed subjects are 7 years older than the non-depressed control group on average. However, the range of the sample is restricted to the ages 20 to 65. A systematic influence of age in this period of life on the effects between the patient groups is unlikely.

It is also important to note that we used a quasi-experimental design, because we wanted to analyze “spontaneous” rumination to prevent induction of experimental artefacts. Therefore, all associations between state and trait rumination and FC are based on between-subject differences. Neither rumination nor depression were induced experimentally and therefore are not controlled and no causality of the effects can be claimed.

To the best of our knowledge, this is the first study comparing the effects of state and trait rumination on the differences in functional connectivity (FC) between depressed and non-depressed subjects. We found that only a subsample of depressed subjects report “spontaneous” rumination during resting-state. FC in the DMN is decreased in depressed subjects compared to non-depressed subjects – an effect that is partly associated with the process of mind-wandering and state/trait rumination. In future studies on the neurophysiological correlates of depressive rumination, the latter should be assessed as a trait- as well as a state-construct, as well as spontaneous and induced rumination.

## Materials and Methods

### Participants

Subjects were recruited from participants in the WikiD-study (clinical trial: NCT02375308) conducted at the Clinic for Psychiatry and Psychotherapy at the University Hospital of Tübingen. All used methods and procedures in this study were in accordance to the current guidelines of the World Medical Associations Declaration of Helsinki. This study was approved by the ethics committee at the University Hospital and University of Tübingen. All subjects gave written informed consent. 89 subjects participated in the study. Five subjects were excluded from data analysis due to an insufficient signal quality (fNIRS data). The sample comprised 60 patients with current MDD diagnosed by clinicians based on the structured clinical interview for DSM-IV (SCID)^44^. 32% of the depressive sample were treated with anti-depressive medication (stable for at least 3 months). The mean score of the Patient Health Questionnaire (PHQ-9) was 14.53 (SD = 3.84, range: 6–23) which can be interpreted as a moderate to severe average symptom severity^45^. The mean score on the Montgomery–Åsberg Depression Rating Scale (MADRS) based on clinical ratings was 21.1 (SD = 5.97, range: 6–34) which corresponds to a moderate symptom severity^46^. In the depressed group, 16.66% of the sample showed a comorbid diagnosis of Persistent Depressive Disorder, 10% had a Specific Phobia, 8.33% had the diagnosis of a Personality Disorder, 5% Social Phobia and 3.33% were diagnosed with a comorbid Panic Disorder. 3.3% of the depressed sample had a main school degree, 16.7% a middle school degree, 46.7% a high-school diploma (German *Abitur*) and 33.3% had a university degree.

Twenty-four healthy controls were additionally recruited. 4.2% of the non-depressed sample had a main school degree, 8.3% a middle school degree, 16.7% a high-school diploma, 12.5% a university of applied science degree and 50% had a university degree. None of the control subjects took anti-depressive medication or reported a life-time diagnosis during the SCID interview. The depressed and non-depressed sample did not diverge in the sex-ratio. However, the control subjects were significantly younger (33 years) than the depressed subjects (40 years). As expected, the two groups differed in their symptom severity measured with the PHQ-9 and MADRS (Table 3), but did not differ with respect to their educational level (p > 0.1, ${\chi }_{(1)}^{2}$ = 1.68). 66.7% and 80% of the non-depressed and depressed group, respectively, had a high educational level (high-school diploma or higher).

**Table 3:** Demographic variables of the depressed and non-depressed group.

Variable	Non-Depressed (n = 24)		Depressed (n = 60)		t/χ²	p
	mean	SD	mean	SD		
Age (years)	33	11.45	40	14.79	t ﻿_(82)_= 2.19	p < 0.05
Sex ratio (f/m)	68%		72%		${\chi }_{(1)}^{2}$ = 0.09	p > 0.1
Antidepressive Medication (%)	0%		32%		${\chi }_{(1)}^{2}$ = 10.02	p < 0.001
MADRS	1.43	1.42	21.1	5.97	t_(82)_ = 15.9	p < 0.001
PHQ-9	2.20	1.77	14.53	3.84	t_(82)_ = 15.0	p < 0.001
RRS	1.79	0.37	2.56	0.39	t_(82)_ = 8.4	p < 0.001
Reported Rumination	8.3%	—	40%	—	${\chi }_{(1)}^{2}$ = 8.0	p < 0.01
Reported Mind-wandering	87.5%	—	48.3%	—	${\chi }_{(1)}^{2}$ = 10.9	p < 0.001
Reported FAF	29.2%	—	41.7%	—	${\chi }_{(1)}^{2}$ = 1.1	p > 0.1
Reported Focus on Body Sensation	29.2%	—	8.3%		${\chi }_{(1)}^{2}$ = 6.0	p < 0.05

MADRS = the Montgomery–Åsberg Depression Rating Scale, PHQ-9 = Patient Health Questionnaire, RRS = Rumination Response Scale, FAF = Fight Against Fatique.

### fNIRS

Hemodynamic changes were measured via fNIRS, an optical imaging method using light in the near-infrared spectrum to measure concentration changes of oxygenated and deoxygenated hemoglobin. The penetration depth and therefore spatial measurement depth of fNIRS is approximately 2–3 cm^47, 48^. Advantages of this method comprise a relatively high temporal resolution, mobile application, insensitivity to movement artefacts, low costs and easy assessment^49^. Importantly, fNIRS has been shown to be a useful and reliable device to measure FC^50–53^. We used a continuous wave, multichannel NIRS system (ETG-4000 Optical Topography System; Hitachi Medical Co., Japan) with a temporal resolution of 10 Hz. To measure parts of the DMN, we placed the probeset over parietal areas covering the precuneus^7^ with reference points Pz, T3 and T4, according to the 10–20 system^54^. The system consisted of 52 channels. Channel positions were located using a neuro-navigation system on a volunteer’s head (Table 4).

**Table 4:** fNIRS channels and related brain areas (estimated based on a neuro-navigational measurement in an exemplary volunteer).

Brain area	Channels
Somatosensory Association Cortex	4, 5, 6, 7, 15, 16, 17, 25, 26, 27, 28, 35, 36, 37
Supramarginal gyrus (part of Wernicke’s area)	2, 3, 8, 9, 12, 13, 18, 19, 23, 30
Angular gyrus (part of Wernicke’s area)	14, 24, 29, 34, 39, 40, 45, 50
Superior Temporal Gyrus	11, 21, 22, 31, 33, 41
V3	38, 46, 47, 48, 49
Fusiform gyrus	43, 44, 51, 52
Middle Temporal gyrus	32, 42
Primary Somatosensory Cortex	1, 20
Subcentral area	10

### Resting-State Measurement

Data was assessed during a 7-minute resting phase in which participants were asked to sit still with eyes closed and let their thoughts flow. After completion of the resting-state measurement, subjects documented what they had done during that time and completed visual analogue scales (VAS) regarding the amount of time they had spent with different processes. Subjects were asked to approximately rate on a scale from 0 to 100% how much time they had spent with a specific process (e.g. being relaxed) during the resting-state measurement (see supplemental material). Four main processes were analyzed: state rumination, mind-wandering, fight against fatigue and focus on sensations. Trait rumination was assessed with the Rumination Response Scale^17^. Additionally, subjects were asked to describe their inner experiences during the resting-state measurement in detail on a blank page – the self-report form. The texts were screened and categorized by two independent raters to assess qualitative measures of processes during resting-state according to qualitative methods: First, self-report forms were analyzed and categories were built and defined until saturation was reached. Second, the most common categories were used to categorize self-report forms by two independent psychologists.

## Data Analysis

### Preprocessing

Data were processed and analyzed using MATLAB R2015b (MathWorks Inc, Natick, USA). After preprocessing, the matlab *NBS toolbox* ^55^, *Wavelab850 toolbox* (http://statweb.stanford.edu/~wavelab/) and *BrainNetViewer toolbox* ^56^ (http://www.nitrc.org/projects/bnv/) were used for analyzing and plotting results. Furthermore, PASW (Version 22) was used for data analysis. Data preprocessing included: bandpass filtering (0.1–0.01 Hz) to minimize high- and low-frequency noise, movement artefact reduction by the algorithm of Cui *et al*.^57, 58^, as well as wavelet-based correction of extreme values^59^ to reduce high amplitude artefacts, with the following settings: Mother wavelet ‘Vaidyanathan’, support = 10, threshold = 0.0001, alpha = 0.1^59^. Afterwards, all signals were visually inspected revealing local artefacts after the described pre-processing in 50% of the subjects. In these cases, channels were interpolated from surrounding channels. If more than 10% of the channels had to be interpolated, subjects were excluded from further analysis (n = 4). Since FC can be significantly influenced by global signal changes^52^, a global signal reduction was performed with a spatial gaussian kernel filter^60^ with a standard deviation of σ = 50. After preprocessing, FC-coefficients were computed and transformed via Fishers r-to-z-transformation^61^.

### Network-Based Statistics (NBS)

Subsequent FC-differences between the diagnostic groups were investigated with Network-Based Statistics^55^. NBS is a statistical method that uses massive univariate testing of a contrast on connectivity matrices and clusters connections that exceed a significance threshold using a breadth first search. The size of the extracted cluster is then tested on significance using permutation tests. Settings for NBS were set as follows: statistical threshold for massive univariate testing t = 2.7, t = 3.0 and t = 3.4, significance level for permutation tests α = 0.05, permutations = 5000, component size = “intensity”. We estimated confidence intervals for the computed p-values of the permutation tests parametrically following Zalesky *et al*.^55^:

1 $$2\times \sqrt{\frac{p(1-p)}{M}}\,{\rm{with}}\,{\rm{M}}={\rm{number}}\,{\rm{of}}\,{\rm{permutations}}{\rm{.}}$$

After using NBS, significant network differences between depressed and non-depressed subjects were searched for hub nodes. To identify these regions, two indices were used: The degree of the nodes and the strength of the FC difference in the connections of these nodes between the diagnostic groups (assessed by different statistical thresholds). The degree of a node is defined as the number of connections of that node with other nodes in the network^62^. Figure 1 shows an overview over the analytical steps.

## Electronic supplementary material

Supplementary Information
