# Supplementary material for: Aberrant functional connectivity in depression as an index of state and trait rumination
Source: Sci Rep. 2017 May 19;7:2174. doi: 10.1038/s41598-017-02277-z (PMC5438394; doi:10.1038/s41598-017-02277-z)
Supplement: Supplementary file 1 — Supplementary Information [file 41598_2017_2277_MOESM1_ESM.pdf]

Aberrant functional connectivity in depression as an index of state and trait rumination

David Rosenbaum[1], Alina Haipt[1], Kristina Fuhr[1,3], Florian B. Haeussinger[1], Florian Metzger[1], Hans-Christoph Nürk[4], Andreas J. Fallgatter[1,2], Anil Batra[1], Ann-Christine Ehlis[1]
[1] Department of Psychiatry and Psychotherapy, University Hospital of Tuebingen, Tuebingen, Germany
[2] Center of Integrative Neuroscience (CIN), Cluster of Excellence, University of Tuebingen, Germany
[3] Department of Clinical Psychology and Psychotherapy, University of Tuebingen, Germany
[4] Department of Diagnostics and Cognitive Neuropsychology, University of Tuebingen, Germany

Supplemental Material

Detailed information on the depressed sample

Amongst the most frequently used medication in the MDD group were Selective Serotonin Reuptake Inhibitors (15% of the sample), Serotonin–Norepinephrine Reuptake Inhibitors (8.3%), Noradrenergic and Specific Serotonergic Antidepressants (5%), Tricyclic Antidepressants (3.4%), Melatonin Agonists (1.7%) and Hypericum perforatum (3.4%). Regarding life-time diagnoses, 8.33% were diagnosed each with PDD and Alcohol Abuse, 6.66% with Panic Disorder, 3.33% each with Social Phobia, Specific Phobia and Bulimia Nervosa and 1.66% had each diagnosis of Obsessive Compulsive Disorder, Posttraumatic Stress Disorder and Anorexia Nervosa.

Additional information on the computation of the state-rumination scale

VAS scales for assessing processes during the resting-state comprised the following items:

Mind-wandering:

1) I felt relaxed.
2) I let my mind flow.

Rumination:

3) I ruminated (in the sense of revolving thoughts).
4) I thought about things I have to do/ tried to make plans.
5) I tried to fight certain experiences.
6) I felt stressed.

Focus on sensations:

7) I felt body sensations.

8) I concentrated on things I hear.

Fight against fatigue:

9) I thought about the duration of the measurement.

10) I needed to fight falling asleep.

Additional information on the rating of the self-report form

To validate the used VAS scales and for reasons of additional information on resting-state processes, we also used a qualitative self-report form. On a blank page, subjects were asked to note the experiences they had during the resting-state measurement. The instruction was as follows:

"Please describe in the following what you did during the resting state measurement and how you felt. You may answer the following questions: What did you feel and think during the measurement? How did you react to your thoughts and feelings? What consequences followed your reactions?"

The texts were screened and categorized by two independent raters to assess qualitative measures of processes during resting-state according to qualitative methods: First, self-report forms were analyzed and categories were built and defined until saturation was reached. The following categories were defined:

- Mind-wandering: The subject expressed to be in a relaxed mood and let his mind flow in an unconstrained way without any focus on a particular subject.
  Example: "I relaxed and let my mind flow."
  Example: "I thought about things that matter to me, but I was not stuck in my thoughts. I liked to let my mind flow."
- Rumination: The subject expressed a repetitive stressful style of thinking about an unfinished concern that leads to the urge of suppressing the inner experience.
  Examples: "I thought about a stressful meeting I had at work, which made me nervous, so I tried to distract myself from that memory." "I thought about an argument with my boyfriend and asked myself what I am doing wrong."
- Focus on body sensations: The subject expressed an attentional focus on their body.
  Examples: "I focused on my breathing." "I felt my body and my heartbeat."
- Mindfulness/Relaxation training: The subject expressed to be in a mindful state (detachment from cognition, concentration on breathing with detached mind) or

to perform some kind of relaxation technique (e.g. progressive muscle relaxation).

Example: “I focused on my breathing and watched my mind in a detached way.”

- Suppression: The subject expressed withdrawal from or suppression of unpleasant inner experiences.
- Boredom: The subject expressed that the resting-state was boring.
- Unfinished business: The subject expressed thoughts about things they will do.
  Examples: “I thought about what I would eat for dinner and decided to eat pizza.” “I thought about the homework I have to do.”
- Thinking about the measurement: The subject expressed thoughts about the given instructions or how their data might look like.
- Fight against fatigue: The subject expressed feeling sleepy or trying not to fall asleep.
- Thoughts about the duration of the measurement: The subject expressed thoughts about the duration of the measurement or counted the time.

Afterwards, the most common categories were used to categorize self-report forms by two independent psychologists.

Influence of cofounders

Regarding effects of other resting-state process variables, there was no effect for the factors “focus on sensations” and “fighting against fatigue”. One reason for this finding may be that the variance for these scales was smaller, since many participants focused on body sensations and felt sleepy at some point of the resting-state measurement.

In contrast to that, the scale for measuring mind-wandering was positively associated with FC in the DMN, as expected (see supplemental material Figure S2). NBS analysis of the factor revealed a significant ($p=.026\pm0.0045$) network with 28 nodes and 39 edges, reflecting higher FC in participants reporting high mind-wandering (see supplemental material Figure S3 and Table S1).

Medication status had no effect on FC-differences between depressed medicated and depressed non-medicated subjects ($p>.1$).

FC properties in the probeset

For the whole sample, FC coefficients in the used probeset showed an expected distribution with high connectivity within DMN regions of the middle parietal cortex and the supramarginal gyrus (SupG) and angular gyrus (AngG). These regions showed – as assumed – low to negative FC with the temporal cortex consisting of the superior temporal gyrus, fusiform gyrus and subcentral area (see figure S1). In addition, the self-reported amount of mind-wandering correlated positively with FC measures (see supplemental material Figure S2) and showed significant network differences between subjects reporting high vs. low mind-wandering within the DMN with hub nodes in the middle somatosensory cortex (SAC) and the SupG (see supplemental material: table S1 and Figure S3).

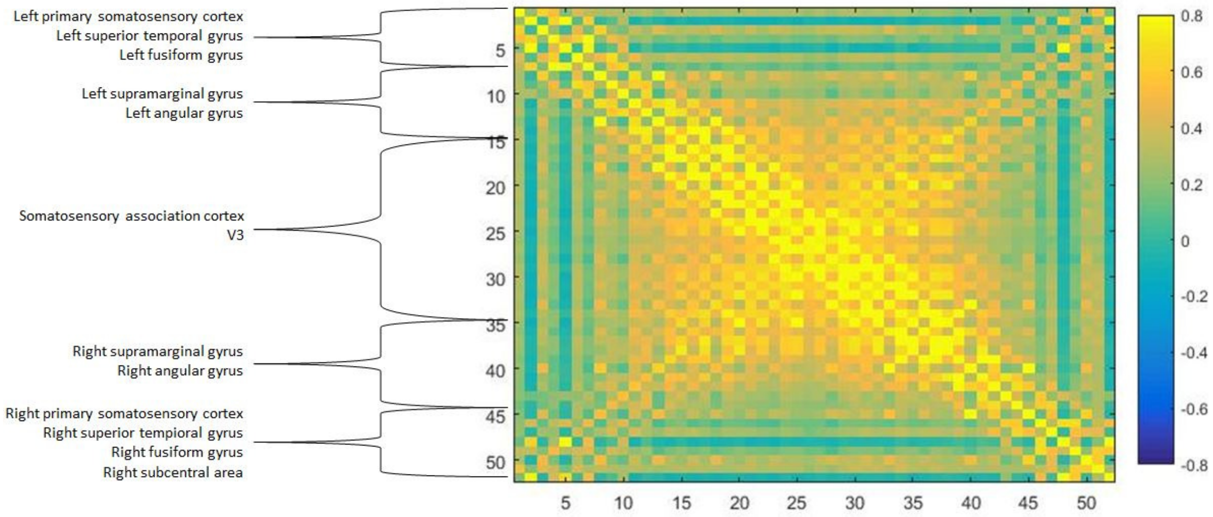

Figure S1. Mean FC of the sample in the different regions of the probeset

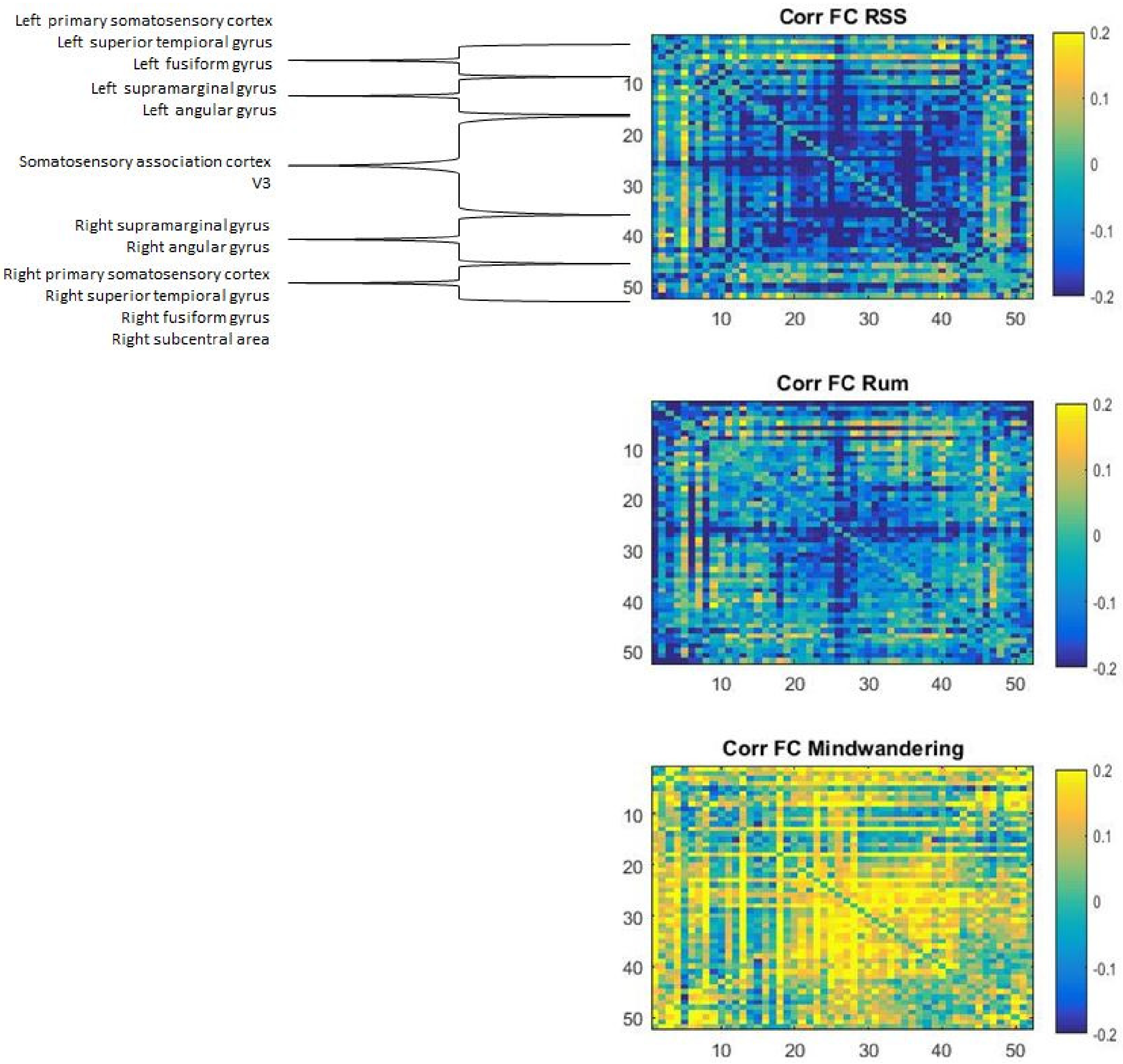

Figure S2. Correlations of trait rumination, state rumination and mind-wanding with FC.

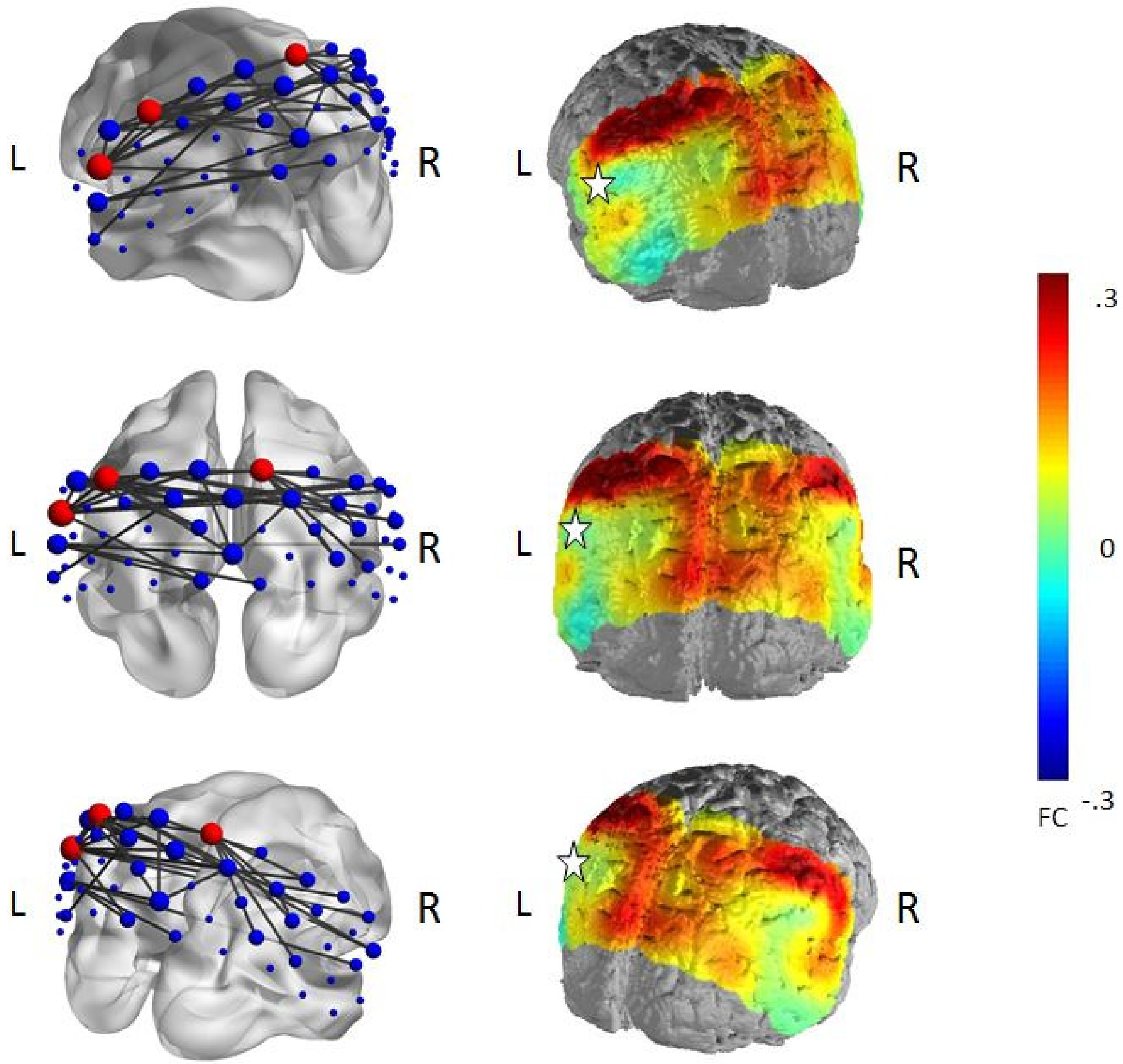

Figure S3. NBS analysis of the main effect for mind-wandering. Left: Significantly hyperconnected network for “high mind-wanderers”. Right: FC maps for the contrast “high vs. low mind-wandering” in the seed region of the left supramarginal cortex. Results of the NBS analysis can be seen in table s1.

Channel	Region	RSS<br>$t_{(82)}$=2.7	Rum<br>$t_{(82)}$=2.8	Mind-wandering<br>$t_{(82)}$=2.9
1	PSC	0	2	0
2	SupG	1	**9**	5
3	SupG	10	0	**8**
4	SAC	10	1	3
5	SAC	6	2	4
6	SAC	10	3	**6**
7	SAC	1	0	1
8	SupG	1	0	2
9	SupG	0	0	1
10	SA	7	0	1
11	STG	1	3	0
12	SupG	2	**13**	**10**
13	SupG	8	1	0
14	AngG	3	0	1
15	SAC	7	0	3
16	SAC	**21**	**6**	4
17	SAC	2	3	3
18	SupG	7	0	2
19	SupG	4	0	1
20	PSC	1	0	0
21	STG	2	0	2
22	STG	0	1	4
24	AngG	2	0	0
26	SAC	3	2	2
27	SAC	3	2	0
28	SAC	2	1	1
29	AngG	3	0	2
30	SupG	1	0	0
31	STG	0	0	1
32	MTG	0	0	1
34	AngG	1	0	0
35	SAC	0	1	0
36	SAC	2	2	0
37	SAC	7	1	4
38	V3	2	2	0
39	AngG	1	0	2
40	AngG	4	0	1
45	AngG	1	0	0
46	V3	2	1	0
47	V3	**18**	1	2
48	V3	3	1	1
49	V3	**12**	0	0
50	AngG	3	0	0
nodes		37	21	28
edges		87	29	39
p-value		.002± .0013	.022± .0041	.023± .0041

Table S1.: Results of the NBS analysis for the main effects of trait-rumination (RSS), state-rumination (Rum) and mind-wandering. Bold numbers are hub nodes.

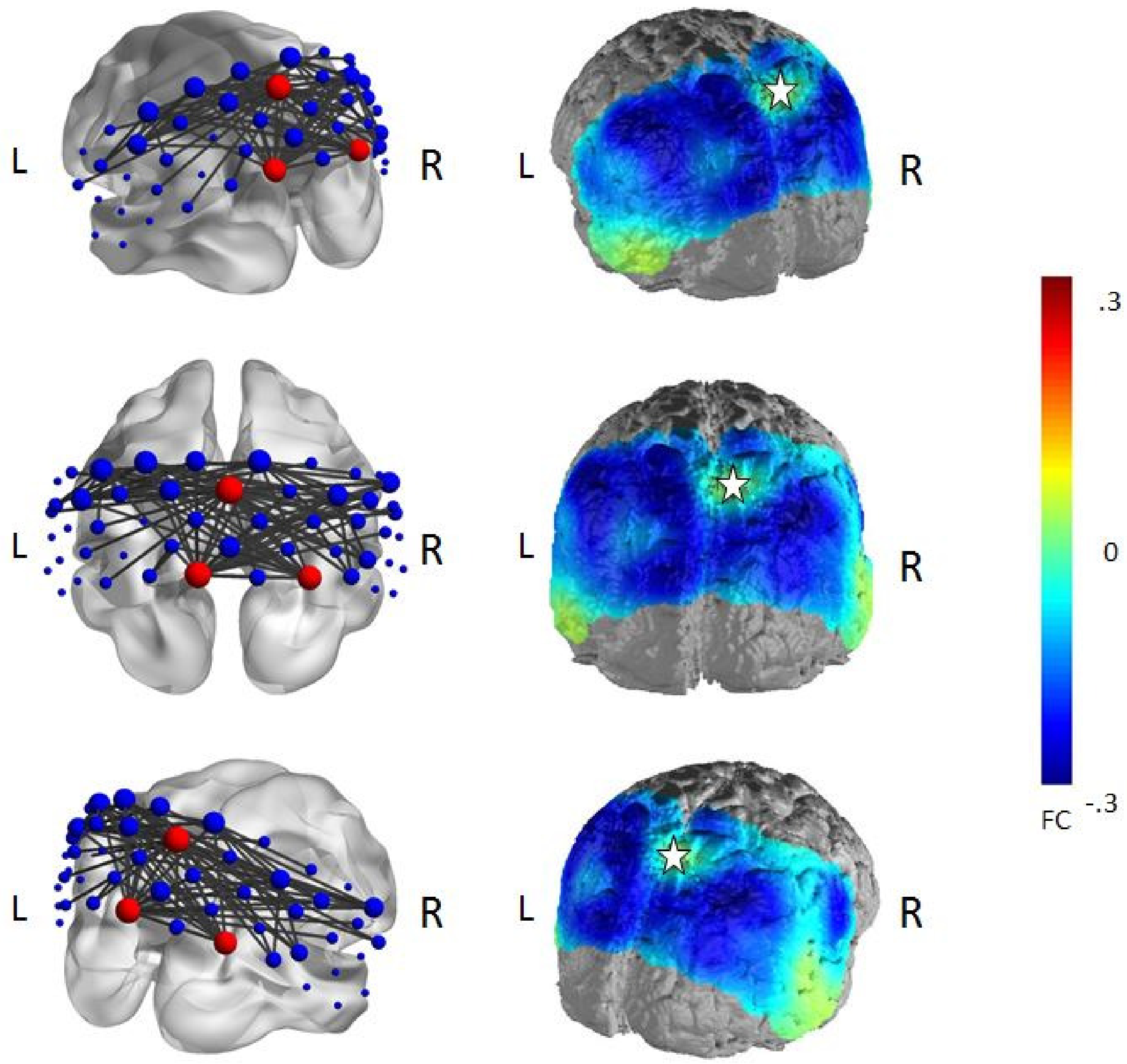

Figure S4. NBS analysis of the main effect for trait rumination. Left: Significantly disconnected network for “high trait-ruminators”. Right: FC maps for the contrast “high vs. low trait-ruminators” in the seed region of the middle SAC. Cold colours indicate higher FC for the low-rumination group. Results of the NBS analysis can be seen in table s1.

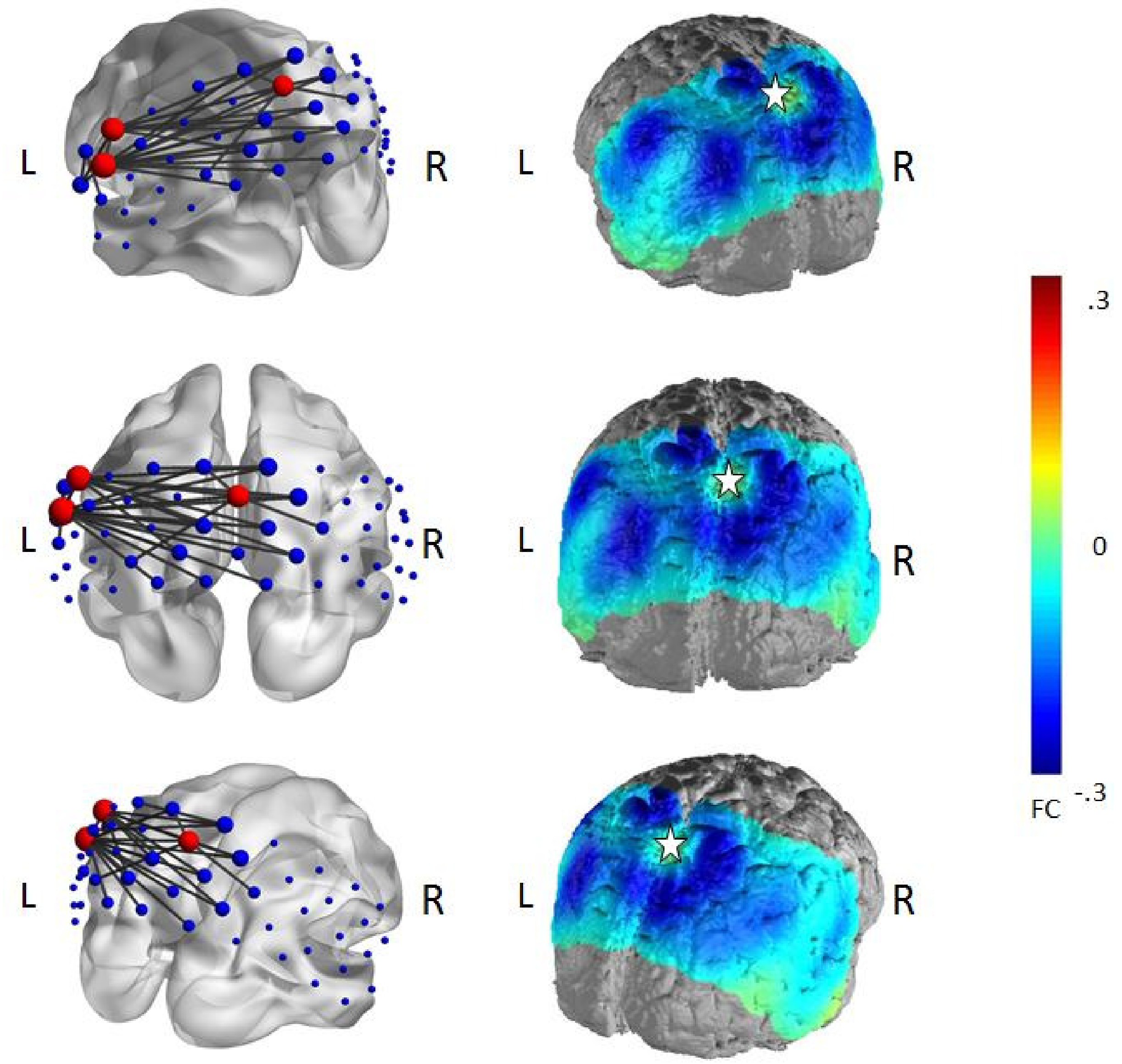

Figure S5. NBS analysis of the main effect for state-rumination. Left: Significantly disconnected network for "high state-ruminators". Right: FC maps for the contrast "high vs. low state-ruminators" in the seed region of the middle SAC. Cold colours indicate higher FC for the low-rumination group. Results of the NBS analysis can be seen in table s1.

	Seed13				Seed4				Seed29			
	RSS		Rum		RSS		Rum		RSS		Rum	
Ch	Rho	p-value	rho	p-value	rho	p-value	rho	p-value	rho	p-value	rho	p-value
1	-0,08	0,469	-0,14	0,197	-0,05	0,653	-0,20	0,062	-0,12	0,258	-0,07	0,530
2	**-0,25**	0,020	**-0,29**	0,008	**-0,31**	0,004	**-0,22**	0,047	**-0,30**	0,006	-0,13	0,244
3	**-0,23**	0,034	-0,17	0,126	**-0,27**	0,014	-0,09	0,429	**-0,29**	0,007	-0,04	0,737
4	**-0,35**	0,001	-0,14	0,191	-	-	-	-	**-0,36**	0,001	-0,17	0,132
5	**-0,40**	0,000	-0,15	0,160	**-0,25**	0,022	-0,06	0,571	**-0,23**	0,033	-0,06	0,601
6	**-0,41**	0,000	**-0,22**	0,040	**-0,28**	0,009	**-0,25**	0,023	**-0,34**	0,002	-0,15	0,170
7	**-0,40**	0,000	-0,14	0,211	**-0,22**	0,041	-0,20	0,066	**-0,25**	0,024	-0,03	0,814
8	**-0,25**	0,022	-0,05	0,648	**-0,26**	0,019	-0,17	0,122	**-0,30**	0,005	-0,04	0,736
9	-0,20	0,065	0,05	0,664	-0,19	0,076	-0,15	0,170	**-0,23**	0,036	0,05	0,637
10	**-0,36**	0,001	-0,17	0,118	-0,14	0,191	-0,10	0,369	**-0,23**	0,037	-0,12	0,257
11	-0,12	0,259	-0,21	0,057	-0,13	0,253	**-0,28**	0,010	-0,08	0,468	-0,11	0,308
12	-0,02	0,830	-0,01	0,964	**-0,27**	0,012	-0,12	0,260	-0,12	0,293	0,05	0,666
13	-	-	-	-	**-0,35**	0,001	-0,14	0,191	**-0,30**	0,006	-0,02	0,836
14	**-0,29**	0,007	-0,08	0,467	**-0,27**	0,012	-0,11	0,303	-0,21	0,052	-0,09	0,397
15	**-0,25**	0,022	-0,11	0,315	**-0,30**	0,006	-0,10	0,371	**-0,22**	0,042	-0,12	0,282
16	**-0,42**	0,000	**-0,29**	0,007	**-0,28**	0,009	-0,08	0,447	**-0,30**	0,005	**-0,26**	0,017
17	**-0,23**	0,034	-0,16	0,134	**-0,28**	0,009	-0,21	0,051	-0,15	0,184	-0,18	0,102
18	**-0,33**	0,002	0,02	0,891	**-0,31**	0,004	-0,11	0,306	**-0,30**	0,006	-0,10	0,383
19	-0,21	0,053	-0,04	0,749	**-0,28**	0,010	-0,09	0,432	-0,19	0,087	-0,09	0,401
20	-0,07	0,500	0,17	0,124	-0,19	0,083	-0,12	0,258	0,00	0,999	0,18	0,105
21	**-0,25**	0,023	-0,04	0,692	-0,15	0,176	**-0,25**	0,021	-0,13	0,243	-0,06	0,581
22	0,01	0,916	0,10	0,365	-0,07	0,534	-0,13	0,236	-0,06	0,617	0,00	0,988
23	-0,10	0,372	-0,02	0,872	-0,07	0,527	0,07	0,548	0,06	0,583	0,10	0,371
24	-0,19	0,087	-0,14	0,212	**-0,25**	0,020	0,01	0,933	-0,11	0,334	-0,08	0,464
25	-0,13	0,240	-0,08	0,496	**-0,24**	0,027	0,01	0,952	-0,15	0,175	-0,07	0,515
26	-0,18	0,111	-0,08	0,453	-0,20	0,068	-0,01	0,904	-0,17	0,114	-0,04	0,717
27	-0,16	0,143	-0,11	0,327	**-0,22**	0,045	-0,05	0,677	-0,25	0,024	-0,05	0,628
28	-0,20	0,064	-0,01	0,923	**-0,28**	0,011	-0,16	0,157	-0,13	0,228	-0,04	0,748
29	**-0,30**	0,006	-0,02	0,836	**-0,36**	0,001	-0,17	0,132	-	-	-	-
30	**-0,22**	0,048	-0,07	0,551	**-0,21**	0,050	-0,02	0,867	-0,09	0,402	-0,12	0,287
31	0,03	0,774	0,07	0,548	-0,03	0,774	-0,06	0,578	0,07	0,504	0,09	0,415
32	**0,23**	0,038	0,05	0,620	0,18	0,097	0,04	0,738	0,04	0,724	-0,11	0,305
33	0,04	0,730	0,12	0,271	0,01	0,958	0,08	0,467	0,12	0,268	0,10	0,342
34	0,02	0,882	0,00	0,987	-0,13	0,235	-0,12	0,283	-0,02	0,883	-0,08	0,470
35	-0,17	0,133	-0,13	0,249	-0,27	0,014	-0,04	0,706	-0,13	0,254	0,00	0,978
36	-0,19	0,080	-0,06	0,618	-0,20	0,067	-0,06	0,580	-0,21	0,057	-0,05	0,663
37	**-0,25**	0,022	**-0,22**	0,047	**-0,28**	0,010	-0,01	0,896	**-0,25**	0,025	-0,10	0,351
38	**-0,20**	0,074	-0,18	0,104	**-0,26**	0,017	-0,17	0,134	-0,12	0,283	-0,10	0,357
39	**-0,21**	0,054	0,00	0,989	**-0,28**	0,011	-0,15	0,163	-0,17	0,120	0,01	0,900
40	-0,23	0,032	0,03	0,792	**-0,34**	0,001	-0,18	0,101	-0,21	0,057	-0,16	0,136
41	-0,02	0,852	-0,13	0,226	0,06	0,608	0,01	0,932	0,06	0,581	-0,07	0,521

42	0,11	0,329	0,08	0,453	0,19	0,089	0,13	0,250	0,05	0,640	0,04	0,728
43	**0,22**	0,046	0,15	0,163	**0,29**	0,007	0,19	0,076	0,20	0,067	-0,02	0,859
44	-0,05	0,640	-0,02	0,826	-0,05	0,670	-0,10	0,363	0,12	0,289	-0,06	0,603
45	-0,07	0,506	-0,03	0,763	-0,19	0,077	-0,14	0,198	-0,05	0,640	0,11	0,339
46	-0,17	0,121	-0,09	0,414	**-0,27**	0,013	-0,09	0,425	-0,13	0,237	-0,05	0,650
47	**-0,29**	0,008	-0,11	0,314	**-0,30**	0,005	0,01	0,948	**-0,22**	0,042	-0,09	0,427
48	**-0,23**	0,038	-0,06	0,581	**-0,23**	0,033	-0,10	0,387	-0,08	0,451	-0,03	0,766
49	**-0,29**	0,008	-0,14	0,217	**-0,31**	0,004	-0,18	0,104	-0,16	0,145	-0,02	0,861
50	-0,20	0,065	-0,12	0,275	**-0,24**	0,026	-0,14	0,195	-0,26	0,018	-0,09	0,399
51	-0,13	0,257	0,00	0,971	-0,14	0,202	-0,10	0,372	-0,10	0,364	-0,06	0,610
52	0,07	0,533	0,07	0,536	0,09	0,430	0,05	0,647	0,02	0,827	-0,02	0,852

Table S2. Korrelations between FC to the seed regions and state- and trait rumination for the whole sample (N=84). P-values are uncorrected, correlations greater .317 are significant after controlling for Type-I errors.

Correlation of resting-state questionnaire scales and VAS Items

		RRS	Scale Rum	Scale FAF	Scale Mind Wandering	Scale Body
RRS	Spearman's Rho	1,000	,317**	,169	-,431**	,074
	Sig. (2-seitig)		,003	,125	,000	,502
	N	84	84	84	84	84
Scale Rum	Spearman's Rho	,317**	1,000	-,063	-,516**	-,287**
	Sig. (2-seitig)	,003		,569	,000	,008
	N	84	84	84	84	84
Scale FAF	Spearman's Rho	,169	-,063	1,000	-,391**	-,225*
	Sig. (2-seitig)	,125	,569		,000	,039
	N	84	84	84	84	84
Scale Mind Wandering	Spearman's Rho	-,431**	-,516**	-,391**	1,000	-,249*
	Sig. (2-seitig)	,000	,000	,000		,022
	N	84	84	84	84	84
Scale Body	Spearman's Rho	,074	-,287**	-,225*	-,249*	1,000
	Sig. (2-seitig)	,502	,008	,039	,022	
	N	84	84	84	84	84
Relaxing	Spearman's Rho	-,400**	-,546**	-,186	,726**	-,095
	Sig. (2-seitig)	,000	,000	,091	,000	,388
	N	84	84	84	84	84
Mindflow	Spearman's Rho	-,221*	-,070	-,180	,535**	-,316**
	Sig. (2-seitig)	,044	,528	,100	,000	,003
	N	84	84	84	84	84
ToDo	Spearman's Rho	,105	,683**	-,048	-,263*	-,251*
	Sig. (2-seitig)	,342	,000	,662	,015	,021
	N	84	84	84	84	84
Ruminating	Spearman's Rho	,313**	,801**	,125	-,533**	-,195
	Sig. (2-seitig)	,004	,000	,257	,000	,075
	N	84	84	84	84	84
Body Sensation	Spearman's Rho	,090	-,125	-,136	-,316**	,815**
	Sig. (2-seitig)	,415	,256	,218	,003	,000
	N	84	84	84	84	84
Control Myself	Spearman's Rho	,253*	,071	,276*	-,578**	,148
	Sig. (2-seitig)	,020	,519	,011	,000	,179
	N	84	84	84	84	84
Hearing Sounds	Spearman's Rho	,193	-,110	,038	-,431**	,718**
	Sig. (2-seitig)	,079	,319	,731	,000	,000
	N	84	84	84	84	84
Supression	Spearman's Rho	,387**	,464**	,140	-,465**	,033
	Sig. (2-seitig)	,000	,000	,205	,000	,765
	N	84	84	84	84	84
Feeling Stressed	Spearman's Rho	,313**	,534**	,250*	-,649**	-,035
	Sig. (2-seitig)	,004	,000	,022	,000	,749
	N	84	84	84	84	84
Thinking about the duration of the measurement	Spearman's Rho	,369**	,149	,692**	-,582**	,070
	Sig. (2-seitig)	,001	,176	,000	,000	,526
	N	84	84	84	84	84
Fighting with falling asleep	Spearman's Rho	,147	,032	,864**	-,382**	-,217*
	Sig. (2-seitig)	,182	,772	,000	,000	,047
	N	84	84	84	84	84

TableS3. Correlations of the scales and between the scales and the Items of the Resting State Questionnaire.

	Mean		SD		Median		Min		Max	
	HC	MDE	HC	MDE	HC	MDE	HC	MDE	HC	MDE
I felt relaxed	86,46	68,08	11,466	24,171	90,00	70,00	60	5	100	100
I let my mind flow	71,88	71,08	26,777	24,328	80,00	80,00	10	10	100	100
I thought about things I have to do	32,29	38,02	28,589	30,683	27,50	30,00	0	0	90	100
I ruminated	14,33	34,58	17,166	31,211	10,00	22,50	0	0	50	100
I felt sensations of my body	56,67	37,68	31,021	28,132	60,00	30,00	0	0	100	100
I needed to control myself	23,29	33,18	25,506	30,581	15,00	20,00	0	0	80	100
I heard sounds	32,92	25,32	30,321	24,638	22,50	20,00	0	0	100	100
I needed to suppress inner experiences	4,79	19,62	7,442	22,766	0,00	10,00	0	0	30	80
I felt stressed	7,71	16,90	15,250	21,885	0,00	10,00	0	0	60	96
I thought about how long the measurement will last	24,75	36,70	31,489	26,772	10,00	30,00	0	0	100	98
I fought against falling asleep	32,29	42,07	35,201	32,714	20,00	33,50	0	0	100	100

Table S4. Item characteristics of the resting-state VAS scales.

Supplementary Analysis:

As supplementary exploratory analysis, we performed two different analyses that were not directly related to the research question, but gave additional information for the interpretation of the results:

1) We performed a rumination subgroup analysis as defined by the qualitative rumination rating (based on the self-report form) in the MDD group only. To this end, the 40% of the MDD subjects reporting rumination in the self-report form during resting-state were compared with the 60% which did not report rumination in the self-report form.

2) In the main analysis, the RRS total score was used. In a third analysis, we also correlated sub-scores of the questionnaire (brooding and reflection) with the FC scores in the whole sample.

Supplementary Analysis of the qualitative rumination rating in the MDD group only.

The analysis of the 40% of the depressed subjects that reported rumination in the self-report form as compared to the 60% of the depressed subjects that did not report rumination revealed a significant disconnected network (with 36 nodes and 67 edges, $t_{(58)}=2.7$, p=.003).
The network – which showed lower FC in high ruminating subjects – was bilaterally organized and had hubs in the bilateral fusiform gyri and somatosensory association cortex. However, inter-hemispheric disconnections were rare and mediated over central hubs. Effect sizes ranged between d=-.44 to d=-96 within FC to the seed channel in the somatosensory association cortex and between d=-.56 to d=-.94 in the right fusiform gyrus.

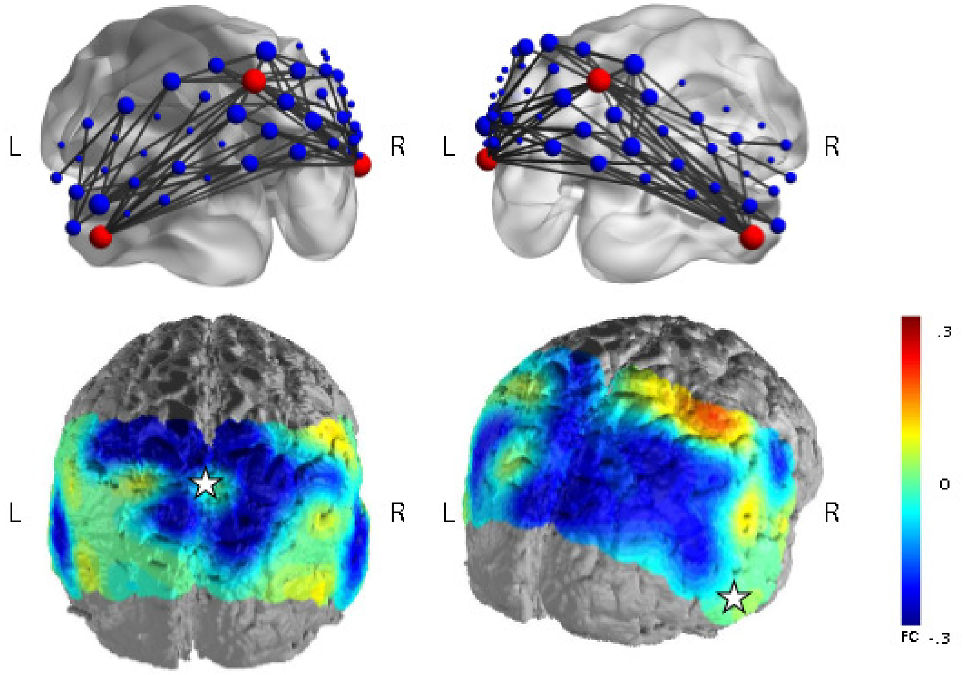

Figure S6. Differences between the subgroup depressed high ruminators and depressed low ruminators according to the qualitative self-report forms. Blue colors indicate reduced FC in high ruminators.

Supplementary Analysis of RRS subscales Rumination and Reflection

As in the analysis of the total RRS score, correlations between FC and the subscale brooding showed negative associations ranging from rho = -.21 to rho = -.36 ($p<.05$ to $p<.001$). The negative relationship between brooding and FC covered areas including the supramarginal gyrus, angular gyrus, somatosensory association cortex, primary somatosensory cortex and the fusiform gyrus. Only the correlation to the right angular gyrus remained significant after controlling for multiple comparisons. In contrast to that, reflection only showed negative correlations with the seed channel 29 and 13. Here, correlations were sparse and located in the somatosensory association cortex and the right supramarginal gyrus. No correlation remained significant after controlling for multiple comparisons

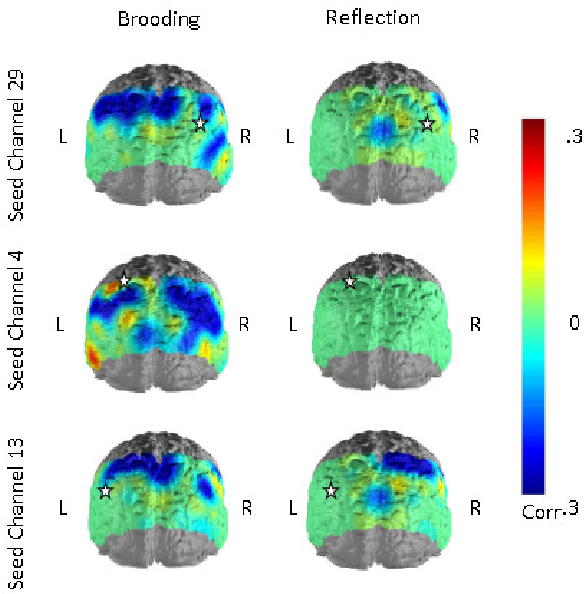

Figure S7. Correlations between seed-channel FC in the depression-related network and subscales of the RRS
